# Traits explain invasion of alien plants into tropical rainforests

**DOI:** 10.1002/ece3.7206

**Published:** 2021-03-25

**Authors:** Decky I. Junaedi, Gurutzeta Guillera‐Arroita, Peter A. Vesk, Michael A. McCarthy, Mark A. Burgman, Jane A. Catford

**Affiliations:** ^1^ School of Biosciences Centre of Excellence for Biosecurity Risk Analysis (CEBRA) The University of Melbourne Parkville VIC Australia; ^2^ Cibodas Botanic Gardens – Research Centre for Plant Conservation and Botanic Gardens Indonesian Institute of Sciences (LIPI) Bogor Indonesia; ^3^ School of Biosciences The University of Melbourne Melbourne VIC Australia; ^4^ Centre for Environmental Policy Imperial College London London UK; ^5^ Department of Geography King’s College London Strand UK

**Keywords:** alien plant naturalization, plant functional traits, Southeast Asia, species invasions, trait‐based invasive risk assessment, tropical rainforest

## Abstract

1. The establishment of new botanic gardens in tropical regions highlights a need for weed risk assessment tools suitable for tropical ecosystems. The relevance of plant traits for invasion into tropical rainforests has not been well studied.
2. Working in and around four botanic gardens in Indonesia where 590 alien species have been planted, we estimated the effect of four plant traits, plus time since species introduction, on: (a) the naturalization probability and (b) abundance (density) of naturalized species in adjacent native tropical rainforests; and (c) the distance that naturalized alien plants have spread from the botanic gardens.
3. We found that specific leaf area (SLA) strongly differentiated 23 naturalized from 78 non‐naturalized alien species (randomly selected from 577 non‐naturalized species) in our study. These trends may indicate that aliens with high SLA, which had a higher probability of naturalization, benefit from at least two factors when establishing in tropical forests: high growth rates and occupation of forest gaps. Naturalized aliens had high SLA and tended to be short. However, plant height was not significantly related to species' naturalization probability when considered alongside other traits.
4. Alien species that were present in the gardens for over 30 years and those with small seeds also had higher probabilities of becoming naturalized, indicating that garden plants can invade the understorey of closed canopy tropical rainforests, especially when invading species are shade tolerant and have sufficient time to establish.
5. On average, alien species that were *not* animal dispersed spread 78 m further into the forests and were more likely to naturalize than animal‐dispersed species. We did not detect relationships between the measured traits and estimated density of naturalized aliens in the adjacent forests.
6. *Synthesis*: Traits were able to differentiate alien species from botanic gardens that naturalized in native forest from those that did not; this is promising for developing trait‐based risk assessment in the tropics. To limit the risk of invasion and spread into adjacent native forests, we suggest tropical botanic gardens avoid planting alien species with fast carbon capture strategies and those that are shade tolerant.

## Graphical abstract

We demonstrate that alien plant species that spread from Indonesian botanic gardens and naturalize in adjacent native tropical rainforests have different traits to alien species that fail to naturalize. Species with high specific leaf area (SLA), small seeds and long residence times were more likely to naturalize than species that lacked these characteristics. Animal‐dispersed species spread over shorter distances and were less likely to naturalize than species not dispersed by animals. These trait‐based differences hold promise for the development of weed risk assessments specific to the tropics.

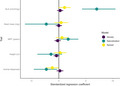

## 1 INTRODUCTION

Trait‐based analyses are central to research in invasion ecology and can inform invasive species risk assessment. If the morphological or physiological features of plants relate to invasion processes, then trait data can enhance invasive species risk assessment by differentiating between alien species of high and low invasion risk (Drenovsky et al., 2012; van Kleunen et al., 2010). There has been considerable research into traits linked with plant invasion, but very few studies have considered invasion in tropical bioregions (Bufford et al., 2015; Peh, 2010; Pysek et al., 2008; Rejmanek & Richardson, 2013; van Kleunen et al., 2010), including southeast Asia (Peh, 2010). Tropical invasion studies can directly inform bio‐security and invasive species management and can test concepts developed in non‐tropical environments. Validation from the tropics could support generalized understanding of invasion causes and mechanisms, thereby assisting invasion science.

It has been proposed that alien plant species in tropical environments would require shade tolerance, fast growth rates, and long‐distance dispersal to become successful invaders (Perkins et al., 2011). The ability to cope with low light conditions (shade tolerance) may be essential for establishment in tropical forests (Poorter, 1999; Bloor & Grubb, 2003) because of limited light availability below the forest canopy. A fast growth rate may help invading species to compete for space and resources when opportunities for recruitment (e.g., forest canopy gaps following disturbance) become available (Martin et al., 2010). Alien species that are able to disperse long distances into intact forests through, for example, animal dispersal may be more likely to spread and become invasive in the tropics because they have a higher probability of reaching sites suitable for recruitment (e.g., canopy gaps) (Dawson et al., 2009; Trakhtenbrot et al., 2005). Dispersal away from the mother plant can also help offspring to (partially) escape enemies (e.g., pathogens, herbivores) (Comita et al., 2014). As opportunities for invasion increase over time (Pyšek et al., 2020), alien species with earlier dates of introduction (i.e., longer minimum residence times) are likely to have greater naturalization success, to be more abundant, and to have spread further than species introduced more recently.

Based on field surveys in and around four botanic gardens in Indonesia, here we examine whether residence time and plant traits linked to light capture (height), physiological performance (SLA as a surrogate for growth rate and shade tolerance), and dispersal (seed mass, dispersal method) relate to alien plant invasion in tropical forests of Indonesia. Southeast Asia is a biodiversity hotspot and global conservation priority (Olson & Dinerstein, 2002), but little research has been conducted into the >400 invasive species present in the region (Peh, 2010), even though invasions pose a threat to regional biodiversity (Gurevitch & Padilla, 2004). Following Dawson et al. (2009), we define naturalized species as exotic species that were planted and present in the garden and were detected in adjacent native forest where they were reproducing; non‐naturalized species are exotic species present in the botanic gardens but not detected in the adjacent native forest. Specifically, we examine the effect of selected traits (specific leaf area (SLA), height, seed mass, and dispersal method) and time since species were planted in the botanic gardens on the following: (a) naturalization probability of 590 exotic species that had been planted across the four botanic gardens and were still present at the time of survey; (b) the abundance (density) of 23 naturalized exotic species detected in adjacent native tropical rainforests; and (c) the distance the 23 naturalized exotics have spread from the botanic gardens.

## 2 MATERIAL AND METHODS

### 2.1 Study sites

Surveys were conducted in four tropical forest ecosystems in Indonesia adjacent to four botanic gardens (Table S1, Figures S2 and S3). These sites are as follows: (a) the eastern slope of Mount Gede‐Pangrango, in close proximity to Cibodas Botanic Gardens (Cibodas); (b) the northern slope of Mount Ciremai, close to Kuningan Botanic Gardens (Kuningan); (c) the southern slope of Mount Slamet, close to Baturraden Botanic Gardens (Baturraden); and (d) the eastern slope of Bukit Tapak, close to Eka Karya Bali Botanic Gardens (Bali). The sites vary in elevation from 800 m to 1,450 m above sea level, and all are characterized by tropical sub‐montane rainforest with dominant species from Lauraceae and Fagaceae (Whitten et al., 1996; Yamada, 1975). Most of the study sites have a relatively wet tropical climate with rainfall of 4,000 to 5,000 mm/year, except for Bali where rainfall is slightly lower (3,000 to 4,000 mm/year). At all sites, rain occurs throughout the year (mostly from November to March) (Whitten et al., 1996). All the gardens were in remote areas, at least 40 km from any large towns. All the forests next to the four botanic gardens are protected areas that restrict human activities. However, local residents and visiting mountain climbers visit limited areas of the forests. Most of the land surrounding the forests consists of remote villages and agricultural land such as paddy fields and vegetable growing areas. Most of the adjacent tropical forests are secondary forests with varying degrees of natural disturbances. Cibodas experiences frequent windstorms (resulting in tree‐fall gaps in the forest) and Kuningan experiences small spot fires during most dry seasons (Abdulhadi et al., 1998). Bali and Baturraden experience relatively few natural disturbances compared with Cibodas and Kuningan.

### 2.2 Vegetation survey and classifying naturalized and non‐naturalized species

We used line transect distance sampling to quantify the extent of invasion in forests surrounding the botanic gardens (Buckland et al., 2007). Surveys ran from the border of the botanic gardens toward the interior of adjacent native forests (Junaedi et al., 2018). The surveys consisted of 4, 6, 4, and 3 transects at Cibodas, Bali, Baturraden, and Kuningan respectively. The mean (and the range) of the transect lengths were 337.5 m (100–450 m) for Cibodas, 150 m (50–150 m) for Bali, 112.5 m (100–150 m) for Baturraden, and 216.7 m (150–350 m) for Kuningan. A transect survey ended once no naturalized aliens were detected beyond 100 m from the last detection location on that transect, and this is the reason why we had different length of transects. The field conditions (land contour), available time, and resources limited the transect sampling availability, and consequently the transect sampling number differed between sites. Total transect length across all sites was 3,350 m. For each alien plant detection, perpendicular distance to the transect was measured with a Laser rangefinder Bosch GLM50 to characterize the detection function. We excluded alien climbers and vines from this study because climber/vine height does not relate to the trade‐off between light access and biomass expenses required to support foliage at height that other growth forms experience (Westoby, 1998).

A total of 590 alien species that had been planted across the four botanic gardens were still present in the gardens at the time of survey. We detected 23 naturalized species across the four study sites (Table S4). We define naturalized species as alien species that exist in the botanic garden collections and were also detected in the forests next to the botanic gardens during the transect surveys. The non‐naturalized species are defined as the botanic gardens alien collections that were not detected in the transect (adjacent forests). Thus, the detection data refer to the inventory data of naturalized alien species that likely originated from botanic gardens alien collections. We collected trait data of naturalized and non‐naturalized species to conduct a “case‐control” study to examine whether the measured traits contributed to the naturalization probability of botanic gardens alien collections. We defined the detected naturalized species as the “risked group” and non‐naturalized species as the “non‐risked group.” We measured and collected trait data from 78 randomly selected non‐naturalized species (from a total available 567 non‐naturalized species) (Table S5); we were unable to measure traits of all 567 non‐naturalized species because of time and resource limitations. Our sampling was stratified by botanic garden to ensure it captured alien species from all four sites. Our decision about sample size (i.e., number of species to survey) was based on statistical power. We calculated the sample size in R (R Core Team, 2013) using package powerMediation (Qiu, 2017). We assumed that the event rate (naturalized aliens) at the mean of the continuous independent variable is 0.3. We set the type I error rate at 0.05, and the power to 0.8. The sampling size calculation indicated that we needed data for 78 randomly selected non‐naturalized species to represent the pool of 567 non‐naturalized species. The random sampling covered 36 out of 127 families of alien botanic gardens collection. Based on the Angiosperm Phylogeny Group IV (APG IV) (The Angiosperm Phylogeny Group et al., 2016), the conducted random sampling consisted of 22 orders (21 angiosperm + 1 gymnosperm) from a total of 41 orders (40 angiosperm + 1 gymnosperm) of botanic garden alien collections (Figure S6). Most of the orders of the naturalized aliens (11 out of 12) were also represented in the random sampling, and the genera covered in the random sampling were closely related to the naturalized genera except for *Piper* (one naturalized species, *Piper aduncum*) (Figure S7). The total sample sizes were 913 and 195 for naturalized and non‐naturalized individuals, respectively. We obtained fewer individuals for non‐naturalized species because numbers of non‐naturalized species were limited (i.e., only 2–10 individuals of the non‐naturalized species were planted in the botanic gardens, thus limiting the number of individuals we could sample).

### 2.3 Density of naturalized species in the adjacent native rainforests and their distance from the gardens

We used the line transect distance sampling data to estimate the density of naturalized alien species in adjacent forest (Buckland et al., 2001). As the length and number of transects were different in each study site due to different field conditions, we focused on density (individuals per ha) rather than total abundance. To enable density estimation for alien species that were rarely detected, we built a hierarchical model for the detection function where traits influence detectability, as in Junaedi et al. (2018). These traits were leaf area, leaf shape, and plant heights (File S8).

We measured the distance from the edge of the garden to all naturalized aliens that were detected. We defined a species’ distance of naturalized plants from the gardens as the median distance between the detection location and the gardens across all individuals of the species.

### 2.4 Plant traits

We gathered data on the following traits: specific leaf area (SLA), height, seed mass, dispersal method, and residence time (Table 1) based on Pérez‐Harguindeguy et al. (2013). Trait data collection involved a combination of measurements in the field and extracting data from the following repositories: Global Biodiversity Information Facility (gbif.org), Hawaiian Ecosystem at Risk (hear.org), efloras.org, and Biodiversity Heritage Library (biodiversitylibrary.org) (plant description only, did not include herbarium collections).

**Table 1:** List of measured traits and hypothesized responses across three proxies of invasion processes: probability of naturalization from botanic gardens, density, and their spread distance from botanic gardens into adjacent forests. Naturalized: naturalization probability, Density: local density, Spread: spread distance. (~) indicates varied correlation (might be negatively or positively correlated) between corresponding traits and naturalization, density and/or the spread of alien plant species

Trait	unit	Explanation	Expected correlation	References
Specific leaf area (SLA)	mm^2^/mg	Ratio between leaf area and its oven‐dry mass	Naturalized (+)	Martin et al. (2009); Van Kleunen et al. (2010)
			Density (~)	Jäger et al. (2013); Cornwell and Ackerly (2010); Gibert et al. (2016)
			Spread (−)	Daehler (2003)
Seed mass	mg	Total dry weight of 1,000 seeds	Naturalized (−)	Hamilton et al. (2005); Gosper and Vivian‐Smith (2010); Dawson et al. (2011)
			Density (~)	Rejmanek and Richardson (1996)
			Spread (~)	Thomson et al. (2011)
Height	m	The shortest vertical distance between the top leaf (excluding inflorescences) and the ground	Naturalized (+)	Van Kleunen et al. (2010)
			Density (+)	Hamilton et al. (2005); Cornwell and Ackerly (2010)
			Spread (+)	Thomson et al. (2011)
Dispersal method	Binary	Animal dispersed	Naturalized (+)	Swarbrick (1993); Dawson et al. (2011)
			Density (~)	Willson et al. (1989); Lloret et al. (2004)
			Spread (+)	Swarbrick (1993)
Minimum residence time	Years	Number of years since a species was first known to be present in the botanic gardens to the date of the survey	Naturalized (+)	Daehler (2009)
			Density (+)	Crooks (2009)
			Spread (+)	Crooks (2009)

We measured SLA for every individual of the naturalized alien species we detected (a total of 23 species and 913 individuals) and for non‐naturalized aliens (195 individuals with the average and minimum number of individuals per species being 6 and 2, respectively). SLA values at the individual level were averaged (without transformation), and then, we calculated the mean value within each species to obtain mean SLA values at the species level. For SLA data of non‐naturalized aliens, we sampled shaded leaves instead of fully sun exposed as suggested by Pérez‐Harguindeguy et al. (2013) to avoid bias toward environmental differences between shaded forests and open areas of botanic gardens. We did, however, check the relationship between the SLA of leaves grown in the shade versus full sun, and potential effects on model results (see Section 3.1).

Plant height (m) was measured following the method suggested by Pérez‐Harguindeguy et al. (2013). Not all height data were obtained from direct measurement during surveys due to measurement difficulties in the field. Direct measurements were conducted for 40% of the species (40/102 species). All of the 40 directly measured species were mature individuals and were in relatively common environmental conditions. For the remaining 62 species, we collected median height data from botanical descriptions (e.g., http://www.efloras.org/ and http://hear.org) and then calibrated these data using data we collected in the field for the other 40 species. We did this calibration using the following process: First, we plotted the average of heights from direct measurement (39 species) against the median height value from the databases of the same 39 species (Figure S9). Then, we fitted a model to describe the relationship between these two sources of information (direct measured height data and database height data). We chose the model with best fit based on *r* ^2^ values (Table S10). Then, we “calibrated” the height of the remaining 62 alien species based on the fitted model, using the median height data from databases as the predictor variable. The scatter plot between the model prediction and the real data presented in Figure S11.

We did not measure seed mass directly because of limited fruit availability in the study sites during the survey period. Secondary seed mass data (mg) for naturalized and non‐naturalized aliens were obtained from the Kew Seed Information Database (<http://data.kew.org/sid/>). When we did not find seed mass data for a species in the databases (33/102 species), we used the average data of the corresponding genus (29 species) or family (4 species) from the same database, using a minimum of 30 other species.

We included dispersal method, origin, and growth form as categorical variables. We categorized dispersal method as whether the plants are dispersed by animals or not. We focused on animal dispersal because animals are suggested as important vectors for tropical invasion (Dawson et al., 2011; Swarbrick, 1993). The origin of aliens denotes whether the species is native to a tropical or non‐tropical region. We classified species into tropical versus non‐tropical categories because we expected that tropical plant species would be more likely to naturalize in tropical forests than non‐tropical species because of habitat suitability (the study region is tropical). Finally, we classified growth form of alien plants into herbs, shrubs, and trees (we excluded other growth forms, including ferns and vines, due to very low detection rates). Data on dispersal method, origin, and growth form were collected from databases, including <http://data.kew.org/sid/>, <http://www.ars‐grin.gov/>, <http://www.gbif.org>, <http://www.pfaf.org/>, and <http://www.hear.org/pier/>.

Information on minimum residence time, that is, the number of years elapsed since the species was first known to be present in the botanic gardens to the date of the survey was obtained from botanic gardens’ catalogue collections (Cibodas: 1930, 1963, 1977, and 1988; Bali: 1989, 1999, and 2006) and planting date official records (Kuningan and Baturraden). We used minimum residence time in our analyses to account for the potential lag time between the introduction and establishment of an alien species. The complete dataset of all traits for all measured species is presented in Data S1.

### 2.5 Data analysis

We conducted three regression‐based analyses to assess how species traits relate to the probability of naturalization of aliens (model 1), the estimated density of naturalized aliens (model 2) and distance spread from gardens of naturalized alien species (model 3). Prior to analyses, we assessed correlations between explanatory variables (traits) and excluded growth form because it was highly correlated with height (Pearson correlation = 0.76). The final set of traits considered for analysis included: SLA, seed mass (SM), height (H), minimum residence time (MRT), and dispersal method (DM). All explanatory variables were continuous, except for dispersal method, which was binary. We did not include propagule pressure in the models because Indonesian botanic gardens limit the number of planted collections (including alien collections) to five individuals; therefore, the number of individuals for each alien species in the collections did not differ greatly.

We limited the number of predictors within a model to a maximum of five to avoid over‐fitting. We fitted the models using JAGS (Plummer, 2003), called from R with package jagsUI (Kellner, 2015). All analyses were conducted in R (R Core Team, 2013) within the Bayesian framework of inference. We ran 3 MCMC chains and drew 1,000,000 samples per chain, omitting the first 500,000 as a burn‐in. We used the Gelman‐Rubin R‐hat diagnostic to assess the convergence of the MCMC chains (Gelman & Rubin, 1992) and assumed convergence when R‐hat values were smaller than 1.1. We used vague priors for all the parameters. Computer code for all three analyses is available in the Supplementary material (File S12, S13, and S14).

For the first analysis (model 1), we used logistic regression to test whether traits could distinguish naturalized from non‐naturalized species. The logistic regression model was:

(1) $$p_{i}=e^{g_{i}}/(1+e^{g_{i}}),$$

where *p_i_* is the estimated probability that alien species *i* became naturalized, and the linear predictor is *g_i_* = *a*_0_ + *ß_1_*SLA_i_ + *ß_2_*DM*_i_* + *ß_3_*MRT*_i_* + *ß_4_*H*_i_* + *ß_5_*SM*_i_* + *ɛ_i_*. We did not calculate the naturalization probability of botanic gardens alien collections per se*,* because we did not collect the data for all non‐naturalized alien collections in four botanic gardens. Instead, we estimated a relative naturalization probability based on detected naturalized aliens and 78 sampled non‐naturalized alien species, and focused on the effect of traits on this relative naturalization probability.

**Figure 1:**
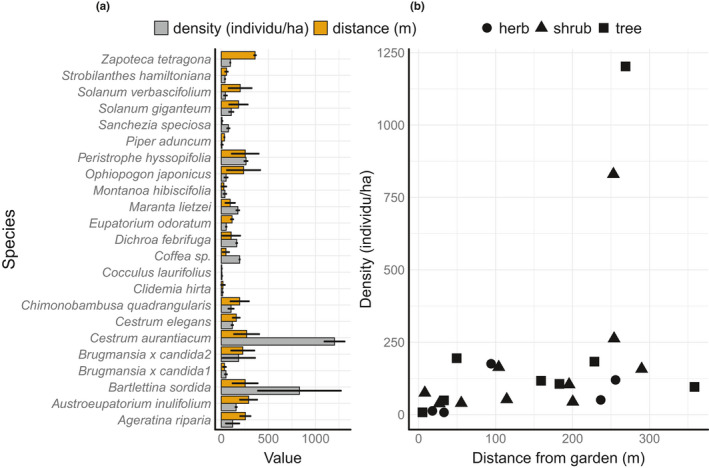
(a) Median distance spread from the botanic gardens of the 23 naturalized alien species (metres) and their density (individuals/hectare) (left panel) and (b) distance from garden of naturalized alien species versus density (right panel)

Our second and third analyses (models 2 and 3) used multiple linear regression. In model 2, we examined the relationship between traits and the estimated density of naturalized aliens. Estimated densities ranged from 14 to 3,551 individuals/ha. The distribution of density estimates was right skewed, not normally distributed (Figure 1). We log‐transformed (base 10) the estimates prior to analysis. In model 3, we examined the relationship between traits and the distance spread from gardens of naturalized aliens, log_10_‐transformed. We did not include location factors in all models since there were only 23 alien species naturalized, and we only detected up to 5 naturalized alien species in two botanic gardens (Baturraden and Kuningan). Model 2 and model 3 are formulated as:

(2) $${\hat{A}}_{i}=a_{0}+{ß}_{1}SLA_{i}+{ß}_{2}DM_{i}+{ß}_{3}MRT_{i}+{ß}_{4}H_{i}+{ß}_{5}SM_{i}+\varepsilon_{i},$$

(3) $${\hat{S}}_{i}=a_{0}+{ß}_{1}{\text{SLA}}_{i}+{ß}_{2}{\text{DM}}_{i}+{ß}_{3}{\text{MRT}}_{i}+{ß}_{4}H_{i}+{ß}_{5}{\text{SM}}_{i}+\varepsilon_{i},$$

where *Â_i_* is the average of the log_10_ of predicted density for species *i*, *Ŝ_i_* the average of the log_10_ of median distance spread from gardens by naturalized alien species *i* (across all transect surveys to all gardens), and *ɛ_i_* are normally distributed errors (*ɛ_i_* ~ *N* (0, *σ*)). We fitted all models (1, 2, and 3) with the predictors standardized, obtained by subtracting the mean and dividing by two standard deviations (Gelman, 2008).

## 3 RESULTS

**Figure 2:**
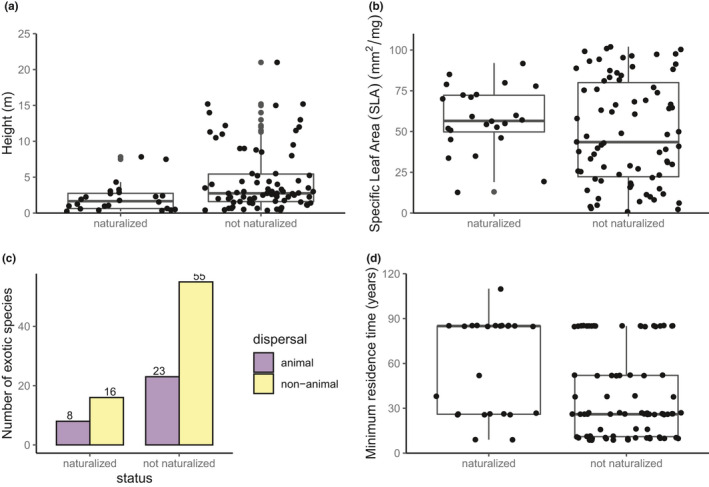
(a) Height, (b) specific leaf area, (c) dispersal method, and (d) minimum residence time of naturalized and non‐naturalized alien species. Dataset contains 102 alien (exotic) species (24 naturalized and 78 non‐naturalized species) and consists of 1908 individual measurements (912 naturalized and 996 non‐naturalized replicates). Boxplots show the median (thick line), inter‐quartile range (box) and lower and higher values outside interquartile range (whiskers extend to no more than 1.5 times the interquartile range beyond the box)

The 23 naturalized and 78 non‐naturalized alien species differed in their trait values. The naturalized species tended to be shorter (mean height 2.1 m versus 4.6 m) and had higher SLA (mean 38.0 mm^2^/mg versus 13.7 mm^2^/mg) than the non‐naturalized species (Figure 2a and b). Twenty three of 78 non‐naturalized species and 4 of 23 naturalized species were trees. Animal dispersal was slightly more common among naturalized species; 33% of naturalized species were animal dispersed compared with 30% of non‐naturalized species (Figure 2c). Minimum residence times (MRT) greater than 30 years were more common among naturalized species while mean residence times less than 50 years were more common among non‐naturalized species (Figure 2d).

### 3.1 Estimated naturalization probability

**Figure 3:**
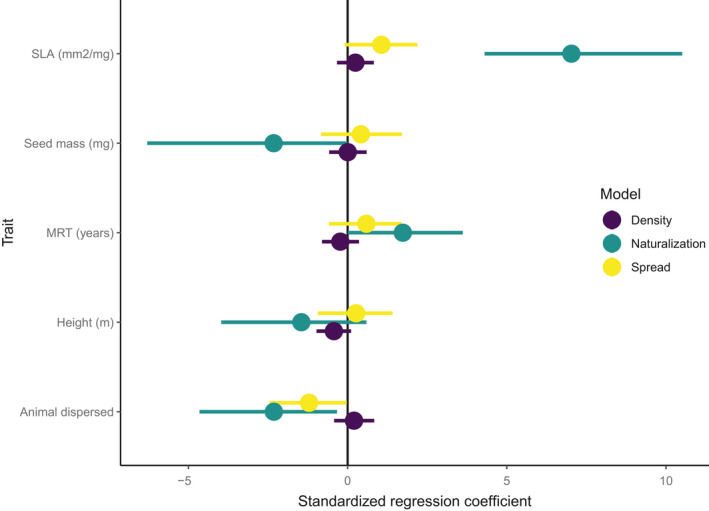
Regression coefficients for alien species traits (standardized) explaining: relative naturalization probability of alien species (model 1, green), naturalized species density (model 2, purple), and naturalized species spread distance from botanic gardens (model 3, yellow). The dots indicate the coefficient means, and horizontal lines represent the 95% credible intervals. Model 1 is a logistic regression model while models 2 and 3 are linear regression models. MRT: minimum residence time; SLA: specific leaf area. Traits with significant relationships (CI not crossing the zero vertical black line) with the response variables are SLA, seed mass, minimum residence time, and animal dispersal for the naturalization model (green) and animal dispersal for the distance spread model (yellow)

The probability of an alien species escaping from the botanic gardens and becoming naturalized in adjacent forests increased with SLA and minimum residence time (Figure 3). For every standardized unit increase in SLA and residence time, the log odds of an alien species naturalizing increased by 7.03 and 1.73, respectively. The positive relationship between SLA (of shaded leaves) and naturalization probablity (Figure 3) held even when we used SLA values from leaves grown in full sun (Figure S15, Table S16), sugesting that the trends we found are robust to sun/shade status of the leaves.

**Figure 4:**
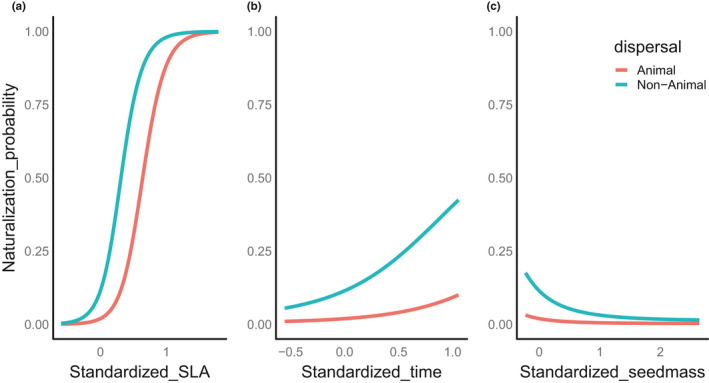
The relationships between: (a) standardized specific leaf area (SLA), (b) standardized minimum residence time, and (c) standardized seed mass and the predicted naturalization probability (model 1) of aliens from botanic gardens for animal‐dispersed aliens and non‐animal‐dispersed aliens

**Figure 5:**
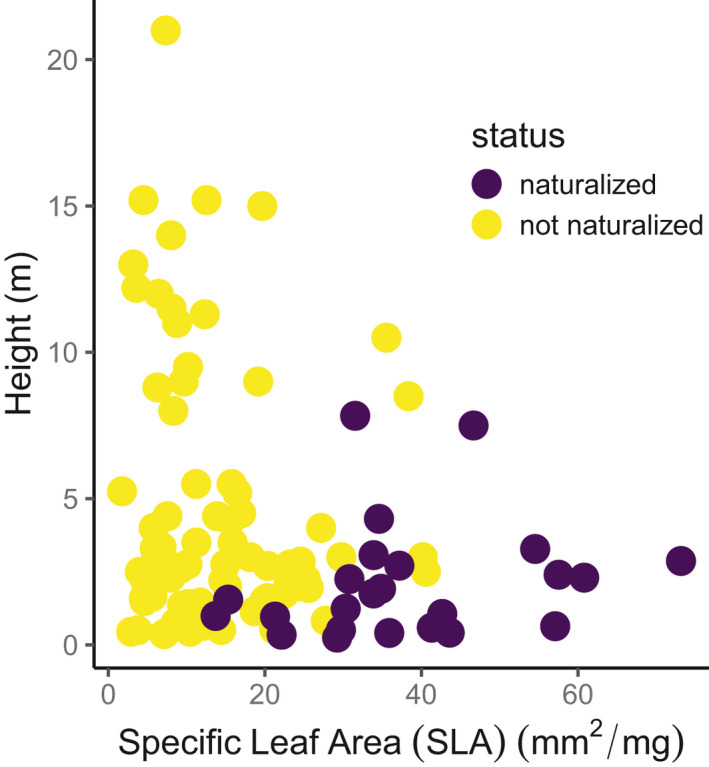
Scatter plot between SLA and height of naturalized (23 species, crosses) and non‐naturalized (78 species, circles) alien species

Animal‐dispersed species and those with higher seed masses were less likely to naturalize than aliens without capacity for animal dispersal and lower seed masses (log odds ratio = −2.317 and −2.323 for animal dispersed and seed mass of alien species, respectively, Figure 3). The predictions from the model of naturalization probability illustrate the dominant influence of SLA (Figure 4). Even though naturalized species were generally shorter than non‐naturalized ones, plant height was not significantly related to species’ naturalization probability when considered alongside other plant traits. Naturalized versus non‐naturalized aliens were clearly differentiated when we plotted their SLA and height together (Figure 5). The tallest naturalized aliens (Figure 1, more than 5 m) that were detected in this study were *Zapoteca tetragona* and *Calliandra callothyrsus*; both are fast‐growing leguminous trees.

### 3.2 Density of naturalized alien species

Our analysis showed no clear evidence of an effect of traits on density of naturalized aliens. It suggested a slight positive correlation between density and SLA, seed mass and time, and negative correlation with height and animal‐dispersal (Figure 3) but these trends were not statistically significant.

### 3.3 Distance spread of naturalized aliens from the gardens

The 23 naturalized aliens spread maximum distances of 5 m to 363 m from the gardens (maximum survey distance from botanic gardens was 450 m). Our model found that animal‐dispersed species were less likely to spread far into the adjacent native forests (Figure 3). Although not significant, there was a positive correlation between species SLA and distance spread. The point estimate corresponds to a ten‐fold increase in distance spread over two standard deviations of SLA.

## 4 DISCUSSION

Our study has shown that particular traits can distinguish alien species that naturalize in native tropical forests from those that do not. Species with higher SLA, longer residence times, smaller seeds and those not dispersed by animals were more likely to escape from botanic gardens and establish self‐sustaining (reproducing) populations in adjacent forests in Indonesia. Among species that successfully naturalized however, the estimated effects of traits on species’ density and distance spread were small or uncertain. This suggests that the five traits examined here are only weakly related to the invasiveness of naturalized alien species, as indicated by distance spread and local abundance (sensu Catford et al., 2016). The one significant exception was dispersal method where non‐animal dispersal was positively linked with distance spread.

### 4.1 Trait‐based relationships

SLA discriminated most strongly between naturalized and non‐naturalized species in the tropical forests in this study. SLA has been linked with species colonization and naturalization (Catford et al., 2019; Dawson et al., 2011) and the presence of alien species in native ecosystems (Penuelas et al., 2010). A high SLA value of established alien species in the tropics has been suggested to indicate their high growth rates (Bufford et al., 2015; van Kleunen et al., 2010) [but see Gibert et al. (2016)] and may facilitate establishment and naturalization of alien species in forest understories. The positive correlation between SLA and growth rate is known to diminish as plants grow larger or older (Gibert et al., 2016). However, SLA provides a useful surrogate for growth rate of alien herbs, shrubs, and small trees because these growth forms are generally short in stature and their life cycles are faster than those of tree species. While tree species outnumber non‐tree species in the four botanic gardens we examined (Figure S19), the majority of naturalized aliens in this study are shrubs and herbs, not trees. Thus, positive correlations between SLA and both naturalization probability and density suggest that fast growth facilitates naturalization of alien species in the tropics. These conclusions are further supported by the clear differentiation between naturalized and non‐naturalized aliens based on SLA and height (Figure 5); among the pool of species examined here, naturalized species had high SLA, and high SLA species were short. If fast growth explains alien species establishment and naturalization, the occurrence of forest gaps will be crucial for alien species naturalization in tropical forests. Forest gaps increase light availability in the forest understory where alien shrubs and herbs can establish and develop. Therefore, forest gaps may also explain the occurrence of naturalized aliens from botanic gardens in adjacent forests. These adjacent forests were exposed to natural disturbances (Werner, 1986), which may have created forest gaps and facilitated the establishment of naturalized aliens from the botanic gardens.

Among out study species, we found that height and SLA were confounded; naturalized species had high SLA and high SLA species tended to be short (Figure 5). Combined with the weak relationship between height and naturalization probability (Figure 3), this suggests that short species are not necessarily at increased risk of naturalization, but rather naturalized aliens had high SLA and tended to be short. We acknowledge that by omitting growth form from the model, it is difficult to assess whether height reflects potential generation time that affects naturalization success. The ability to cope with low light conditions in the forest floor will be crucial for alien species’ persistence in these tropical forests (Martin et al., 2010).

The positive effect of residence time may also reflect the fact that most naturalized aliens are from older botanic gardens in this study (Cibodas and Bali). Fewer species have naturalized from new gardens (Kuningan and Baturraden). There was no relationship between time of planting and type of plants introduced (e.g., for ornamental use or commercial forestry), so we would expect more species to invade from Kuningan and Baturraden in the future.

The lower naturalization probability of animal‐dispersed relative to non‐animal‐dispersed aliens in this study is surprising. Up to 70%–90% of tropical plants are dispersed by animals, and animal dispersal methods are also more common in the wet‐climate tropical forests such as tropical rainforests (Willson et al., 1989). Our finding may reflect the disruption of animal dispersal in these forests, for example, dispersal by primates and frugivorous birds. Humans may have indirectly altered dispersal by impacting the distribution and abundance of animal populations (Dennis & Westcott, 2006). In this study, many naturalized aliens are wind‐dispersed species (e.g., five naturalized Compositae species) and have aggressive vegetative reproduction (e.g., *Chimonobambusa quadrangularis*, *Brugmansia x candida*, *Maranta lietzei,* and *Strobilanthes hamiltoniana*), so that may also explain the trends observed.

### 4.2 Limitations

Plantings in botanic gardens provided us with a useful (unplanned) experiment to examine traits related to alien plant invasion, but our study has several limitations. We only included adult individuals and did not consider the population structure of the alien species. One garden, Cibodas, was the source of most of the naturalized alien species (18 out of 23), and some of these results may have been a consequence of factors unique to that location. For instance, Cibodas holds the largest number of alien plant collections relative to other botanic gardens. We only detected SLA and dispersal method as significant variables when we added botanic gardens as site effect (random effect in generalized linear mixed model and independent variable in multiple logistic regression model) (Table S17). We omitted several possible traits and environmental factors from the model, limiting it to five possible explanatory variables, which we thought would most strongly relate to invasion in this system. Even though we considered seed mass in this study, which means that we partially accounted for potential differences in seed number (Henery & Westoby, 2003), we did not explicitly account for fecundity due to limited data availability. As stated in the trait‐based relationship section (4.1), the context of forest gaps (light factor) is an important consideration for tropical plant invasion. Canopy cover can be used as a proxy for light levels. However, we did not include canopy cover in this study as our focus was on species traits. We recommend including measures of canopy cover (and light intensity and quality) in future studies. Finally, we did not consider phylogenetic non‐independence in this study because the non‐naturalized aliens sampled in this study were all closely related to the naturalized aliens. Phylogenetic information would have been useful for evaluating whether certain clades of non‐naturalized species were over‐represented or not in the random selection process that we used in the study. However, we showed that most of the naturalized taxonomic orders (APG IV) were represented in the random sampling order groups.

### 4.3 Management implications

This study demonstrates that trait‐based models combining field measurement and secondary data can be useful for identifying traits linked with plant invasion in tropical forests. We showed that traits can strongly differentiate naturalized from non‐naturalized alien species, which is promising for developing trait‐based risk assessments in the tropics. Available invasive plant species risk assessment frameworks such as Weed Risk Assessment (Pheloung et al., 1999) or BG‐WRAP (Virtue et al., 2008) incorporate trait information; studies like ours could provide the basis for development of tropic‐specific weed risk assessments.

This study shows that SLA is an important correlate of invasion success of naturalized alien species from tropical botanic gardens; SLA was positively related to the chance of naturalizing and may also be linked to the potential spread rate of naturalized aliens. Further, our study shows that tropical forest understoreys are vulnerable to plant invasion. Thus, we suggest tropical botanic gardens minimize the number of alien species with high SLA in their living collections due to their potential risk of invasion into adjacent tropical forests. Finally, this study suggests that leaf economics (SLA) and dispersal are useful for trait‐based studies in tropical invasion ecology. These traits may also be crucial for invasive plant species management. Management actions such as detections, allocating resources, monitoring, and management evaluation require sufficient understanding of invasion processes and alien species characteristics (Christy et al., 2010; Bogich et al., 2008). Trait‐based risk assessment and early screening that incorporate these traits (SLA, seed mass, height, dispersal method, and residence time) can be useful tools for tropical invasive species management, particularly for predicting which alien plant species that will likely to become naturalized.

## CONFLICT OF INTEREST

Hereby, we stated that there are no potential sources of conflict of interest were involved, considered, and/or included in this manuscript writing, submission, and publication.

## AUTHOR CONTRIBUTIONS

**Decky Indrawan Junaedi:** Conceptualization (equal); Data curation (lead); Formal analysis (equal); Investigation (lead); Methodology (equal); Project administration (lead); Software (equal); Validation (equal); Visualization (lead); Writing‐original draft (equal); Writing‐review & editing (equal). **Gurutzeta Guillera‐Arroita:** Conceptualization (equal); Formal analysis (equal); Methodology (equal); Writing‐review & editing (equal). **Peter A Vesk:** Conceptualization (equal); Formal analysis (equal); Methodology (equal); Validation (equal); Visualization (supporting); Writing‐review & editing (equal). **Michael McCarthy:** Conceptualization (equal); Formal analysis (equal); Funding acquisition (equal); Methodology (equal); Supervision (equal); Validation (equal); Writing‐original draft (equal); Writing‐review & editing (equal). **Mark Burgman:** Conceptualization (equal); Funding acquisition (equal); Methodology (equal); Resources (lead); Software (equal); Supervision (equal); Validation (equal); Writing‐original draft (equal); Writing‐review & editing (equal). **Jane Alexandra Catford:** Conceptualization (equal); Funding acquisition (equal); Methodology (equal); Resources (equal); Supervision (equal); Writing‐original draft (equal); Writing‐review & editing (equal).

## Data Availability

The data set that associated with this study is available at DRYAD (datadryad.org) with https://doi.org/10.5061/dryad.gqnk98sm5.
