# Supplementary material for: Traits explain invasion of alien plants into tropical rainforests
Source: Ecol Evol. 2021 Mar 25;11(9):3808–19. doi: 10.1002/ece3.7206 (PMC8093684; doi:10.1002/ece3.7206)

**Supporting information**

**Table S1.** Study sites and survey locations of line transect distance sampling (Buckland 2001) conducted to detect and estimate the abundance of naturalized botanic gardens’ alien collections.

| **Sampling location** | **Adjacent botanic gardens** | **Mountain**  **slope** | **Coordinates** | **Total transect length (m)** | **Survey time** |
| --- | --- | --- | --- | --- | --- |
| Mount Gede,  West Java | Cibodas  botanic Gardens | Eastern | S 06^0^44.515’  E 107^0^00.290’ | 1350 | April 2015 |
| Mount Tapak,  Bali | Eka Karya Bali  botanic Gardens | Eastern | S 08^0^16.658’  E 115^0^08.985’ | 900 | May 2015 |
| Mount Slamet,  Central Java | Baturraden  botanic Gardens | Southern | S 07^0^18.096’  E 109^0^13.905’ | 450 | June 2015 |
| Mount Ciremai,  West Java | Kuningan  botanic Gardens | Northern | S 06^0^49.519’  E 108^0^24.317’ | 650 | July 2015 |

**
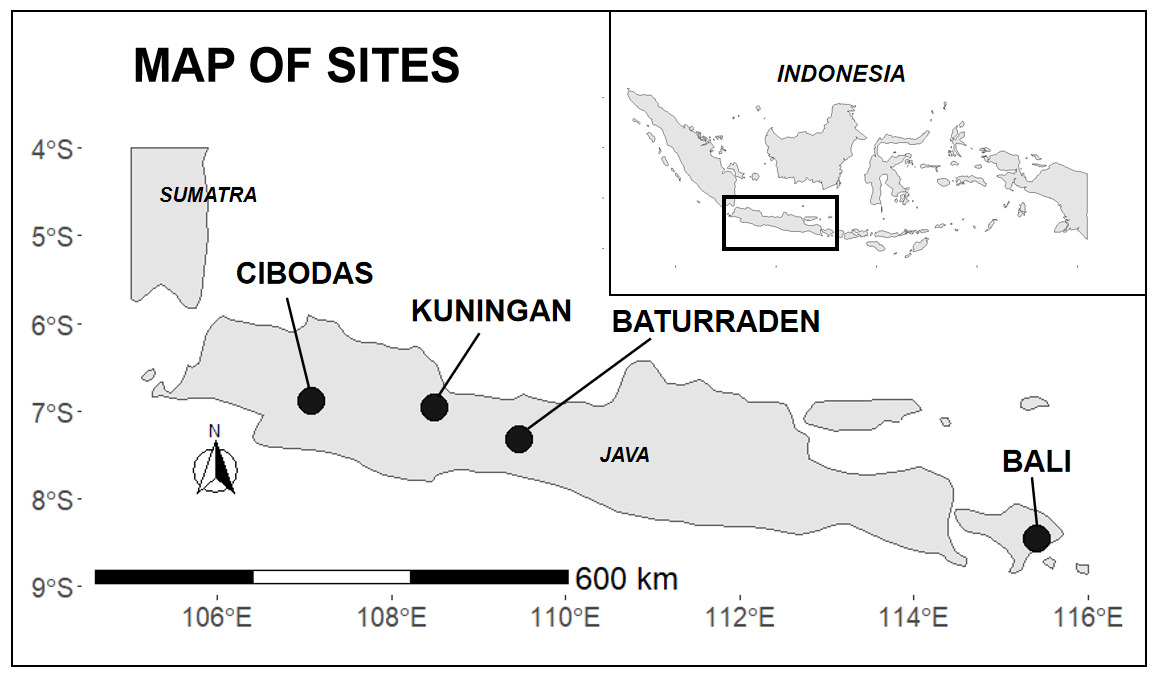
**

**Figure S2.** Map of study sites: Cibodas, Kuningan, Baturraden and Bali. Inset (top-right): map of Indonesia.

**Figure S3.** Map of sampling transect lines (yellow) relative to botanic gardens estimated area (white) and the adjacent forest locations of: A. Cibodas; B. Bali; C. Baturraden; and D. Kuningan. The botanic gardens estimated area did not reflect the precise actual area of botanic gardens and only presented to visualize the spatial context of the transect line relative to botanic gardens. Map generated in Google Maps (https://www.google.com/maps/)
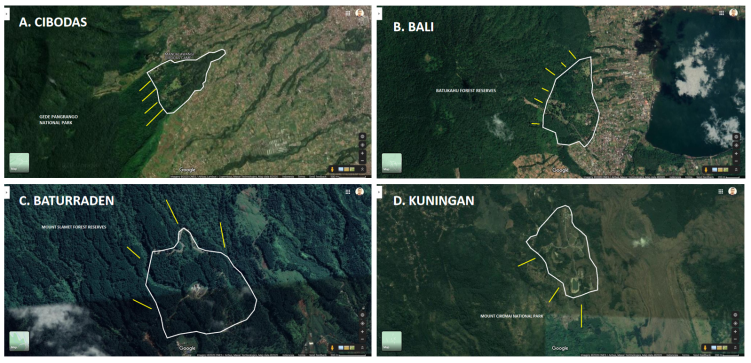
.

**Table S4.** List of 23 naturalized alien collections detected in this study. KBG= Kuningan Botanic Gardens, CBG= Cibodas Botanic Gardens, BRBG= Baturraden Botanic Gardens, and BBG= Eka Karya Bali Botanic Gardens.

| **No** | **Species Name** | **Author** | **Family** | **Garden** |
| --- | --- | --- | --- | --- |
| 1 | *Ageratina riparia* | (Regel) R.M.King & H.Rob. | Compositae | CBG |
| 2 | *Austroeupatorium inulaefolium* | (Kunth) R.M.King & H.Rob. | Compositae | CBG |
| 3 | *Bartlettina sordida* | (Less.) R.M.King & H.Rob. | Compositae | CBG |
| 4 | *Brugmansia x candida2* | Pers. | Solanaceae | CBG |
| 5 | *Cestrum aurantiacum* | Lindl. | Solanaceae | CBG |
| 6 | *Cestrum elegans* | (Brongn. ex Neumann) Schltdl. | Solanaceae | CBG |
| 7 | *Chimonobambusa quadrangularis* | (Fenzl) Makino | Poaceae | CBG |
| 8 | *Clidemia hirta* | (L.) D. Don | Melastomataceae | CBG |
| 9 | *Cocculus laurifolius* | DC. | Menispermaceae | CBG |
| 10 | Coffea sp. | - | Rubiaceae | BBG |
| 11 | *Dichroa febrifuga* | Lour. | Hydrangeaceae | CBG |
| 12 | *Chromolaena odorata* | (L.) R.M.King & H.Rob. | Compositae | BBG |
| 13 | *Impatiens balsamina* | L. | Balsaminaceae | BRBG |
| 14 | *Maranta lietzei* | (E.Morren) C.H.Nelson, Sutherl. & Fern.Casas | Marantaceae | CBG |
| 15 | *Montanoa hibiscifolia* | Benth. | Compositae | BBG |
| 16 | *Ophiopogon japonicus* | (Thunb.) Ker Gawl. | Asparagaceae | CBG |
| 17 | *Peristrophe hyssopifolia* | (Burm.f.) Bremek. (unresolved) | Acanthaceae | CBG |
| 18 | *Piper aduncum* | L. | Piperaceae | CBG |
| 19 | *Sanchezia speciosa* | Leonard (unresolved) | Acanthaceae | BBG |
| 20 | *Solanum giganteum* | Jacq. | Solanaceae | CBG |
| 21 | *Solanum verbascifolium* | L. (unresolved) | Solanaceae | CBG |
| 22 | *Strobilanthes hamiltoniana* | (Steud.) Bosser & Heine | Acanthaceae | CBG |
| 23 | *Zapoteca tetragona* | (Willd.) H.M.Hern. | Leguminosae | CBG |

**Table S5.** List of 78 non-naturalized alien collections randomly sampled in this study. KBG= Kuningan Botanic Gardens, CBG= Cibodas Botanic Gardens, BRBG= Baturraden Botanic Gardens, and BBG= Eka Karya Bali Botanic Gardens.

| **No** | **Species Name** | **Author** | **Family** | **Garden** |
| --- | --- | --- | --- | --- |
| 1 | *Spathodea campanulata* | P.Beauv. | Bignoniaceae | KBG |
| 2 | *Swietenia macrophylla* | King | Meliaceae | KBG |
| 3 | *Acacia mangium* | Willd. | Leguminosae | KBG |
| 4 | *Inga laurina* | (Sw.) Willd. | Leguminosae | KBG |
| 5 | *Albizia saman* | (Jacq.) Merr. | Leguminosae | KBG |
| 6 | *Acanthus montanus* | (Nees) T.Anderson | Acanthaceae | CBG |
| 7 | *Berberis vulgaris* | L. | Berberidaceae | CBG |
| 8 | *Berberis julianae* | C.K.Schneid. | Berberidaceae | CBG |
| 9 | *Bauhinia corymbosa* | Roxb. | Leguminosae | CBG |
| 10 | *Callistemon coccineus* | F.Muell. | Myrtaceae | CBG |
| 11 | *Callistemon citrinus* | (Curtis) Skeels | Myrtaceae | CBG |
| 12 | *Caesalpinia gilliesii* | (Hook.) D.Dietr. | Leguminosae | CBG |
| 13 | *Cupressus sempervirens* | L. | Cupressaceae | CBG |
| 14 | Hippeastrum hybrid | - | Amaryllidaceae | CBG |
| 15 | *Buxus sempervirens* | L. | Buxaceae | CBG |
| 16 | *Asparagus setaceus* | (Kunth) Jessop | Asparagaceae | CBG |
| 17 | *Agrimonia repens* | L. | Rosaceae | CBG |
| 18 | *Canna indica* | L. | Cannaceae | CBG |
| 19 | *Calliandra haematocephala* | Hassk. | Leguminosae | CBG |
| 20 | *Cornus capitata* | Wall. | Cornaceae | CBG |
| 21 | *Cestrum nocturnum* | L. | Solanaceae | CBG |
| 22 | *Clerodendrum hastatum* | (Roxb.) Lindl. | Lamiaceae | CBG |
| 23 | *Buddleja davidii* | Franch. | Scrophulariaceae | CBG |
| 24 | Cupressus sp. | - | Cupressaceae | CBG |
| 25 | *Asparagus aethiopicus* | L. | Asparagaceae | CBG |
| 26 | *Cotoneaster franchetii* | Bois | Rosaceae | CBG |
| 27 | *Callicarpa japonica* | Thunb. | Lamiaceae | CBG |
| 28 | *Cestrum calicynum* | Kunth | Solanaceae | CBG |
| 29 | *Boenninghausenia albiflora* | (Hook.) Rchb. ex Meisn. | Rutaceae | CBG |
| 30 | *Agapanthus africanus* | (L.) Hoffmanns. | Amaryllidaceae | CBG |
| 31 | *Celtis bungeana* | Blume | Cannabaceae | CBG |
| 32 | *Arundo donax* | L. | Poaceae | CBG |
| 33 | *Podocarpus oleifolius* | D.Don. | Podocarpaceae | CBG |
| 34 | *Casuarina equisetifolia* | L. | Casuarinaceae | CBG |
| 35 | *Acacia farnesiana* | (L.) Willd. | Leguminosae | CBG |
| 36 | *Cunninghamia konishii* | Hayata | Cupressaceae | CBG |
| 37 | *Cestrum diurnum* | L. | Solanaceae | CBG |
| 38 | Casuarina sp. | - | Casuarinaceae | CBG |
| 39 | *Callistemon formosus* | S.T.Blake | Myrtaceae | CBG |
| 40 | *Arenga caudata* | (Lour.) H.E.Moore | Arecaceae | CBG |
| 41 | *Casuarina cunninghamiana* | Miq. | Casuarinaceae | CBG |
| 42 | *Ochna kirkii* | Oliv. | Ochnaceae | BRBG |
| 43 | *Kigelia africana* | (Lam.) Benth. | Bignoniaceae | BRBG |
| 44 | *Couroupita guianensis* | Aubl. | Lecythidaceae | BRBG |
| 45 | Markhamia sp. | - | Bignoniaceae | BRBG |
| 46 | *Prunus cerasoides* | Buch.-Ham. ex D.Don | Rosaceae | BRBG |
| 47 | *Anthurium cordatum* | (L.) Schott | Araceae | BRBG |
| 48 | *Caladium bicolor* | (Aiton) Vent. | Araceae | BRBG |
| 49 | Spathiphyllum sp. | - | Araceae | BRBG |
| 50 | *Philodendron panduriforme* | (Kunth) Kunth | Araceae | BRBG |
| 51 | *Philodendron squamiferum* | Poepp. | Araceae | BRBG |
| 52 | *Philodendron melanochrysum* | Linden & Andre | Araceae | BRBG |
| 53 | *Rhododendron mucronatum* | (Blume) G. Don | Ericaceae | BRBG |
| 54 | *Malvaviscus arboreus* | Cav. | Malvaceae | BRBG |
| 55 | *Nerium oleander* | L. | Apocynaceae | BBG |
| 56 | *Scaevola taccada* | (Gaertn.) Roxb. | Goodeniaceae | BBG |
| 57 | *Buxus sempervirens* | L. | Buxaceae | BBG |
| 58 | *Brunfelsia americana* | L. | Solanaceae | BBG |
| 59 | *Lantana camara* | L. | Verbenaceae | BBG |
| 60 | *Cestrum nocturnum* | L. | Solanaceae | BBG |
| 61 | *Eucalyptus robusta* | Sm. | Myrtaceae | BBG |
| 62 | *Eucalyptus globulus subsp. maidenii* | (F.Muell.) J.B.Kirkp. | Myrtaceae | BBG |
| 63 | *Callistemon lanceolatus* | (Sm.) Sweet | Myrtaceae | BBG |
| 64 | *Camellia sasanqua* | Thunb. | Theaceae | BBG |
| 65 | *Gleditsia assamica* | Bor | Leguminosae | BBG |
| 66 | *Costus spiralis* | (Jacq.) Roscoe | Costaceae | BBG |
| 67 | *Berberis truxillensis* | Turcz. | Berberidaceae | BBG |
| 68 | *Hymenocallis littoralis* | (Jacq.) Salisb. | Amaryllidaceae | BBG |
| 69 | *Hydrangea macrophylla* | (Thunb.) Ser. | Hydrangeaceae | BBG |
| 70 | *Solanum pseudocapsicum* var. *diflorum* | (Vell.) Bitter | Soalanceae | BBG |
| 71 | *Justicia carnea* | Lindl. | Acanthaceae | BBG |
| 72 | *Cestrum elegans* | (Brongn. ex Neumann) Schltdl. | Solanaceae | BBG |
| 73 | *Hibiscus mutabilis* | L. | Malvaceae | BBG |
| 74 | *Eugenia uniflora* | L. | Myrtaceae | BBG |
| 75 | *Callicoma serratifolia* | Andrews | Cunoniaceae | BBG |
| 76 | *Rhododendron dilatatum* | Miq. | Ericaceae | BBG |
| 77 | *Oenothera biennis* | L. | Onagraceae | BBG |
| 78 | *Banksia robur* | Cav. | Proteaceae | BBG |

**
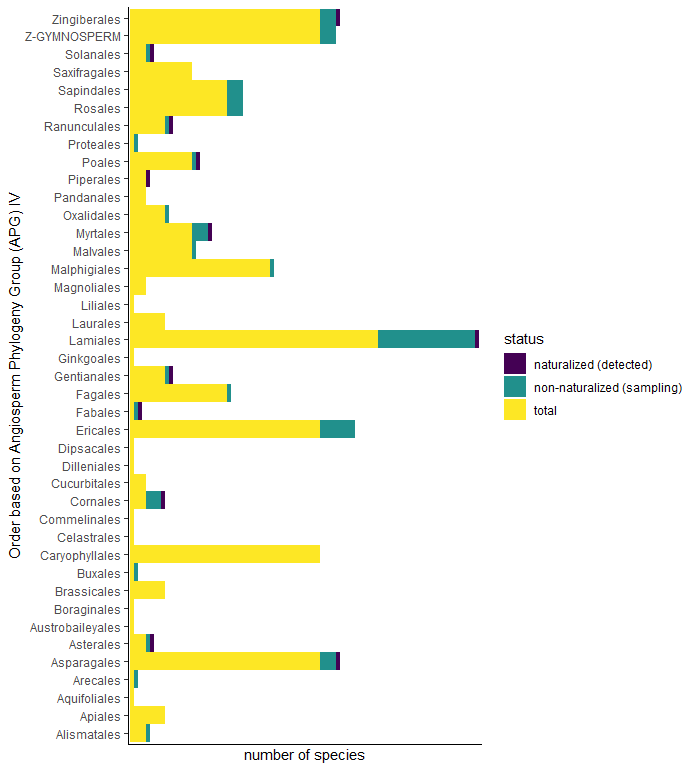
**

**Figure S6.** Barplot of number of families across orders (based on Angiosperm Phylogeny Group, APG IV) (The Angiosperm Phylogeny Group, 2016) of naturalized aliens (dark purple, 12 families and 12 orders), non-naturalized aliens in random sampling (green, 36 families and 22 orders), and total botanic gardens alien collections. (yellow, 127 families and 41 orders).

**
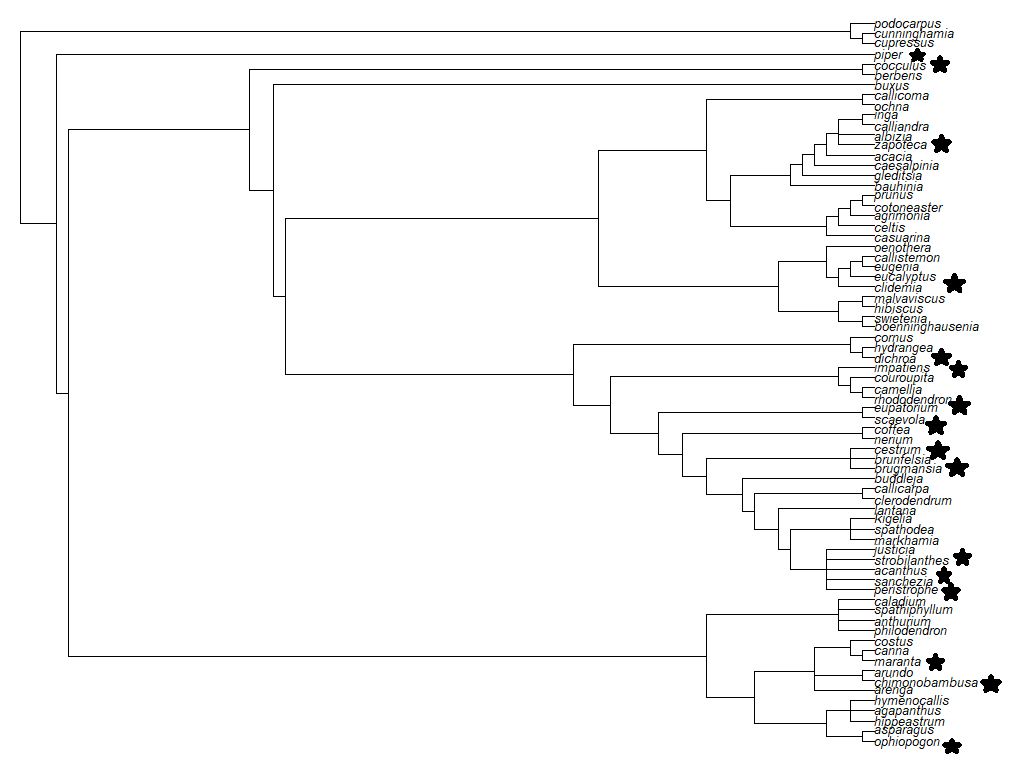
Figure S7.** Phylogeny tree of naturalized and random sampled non-naturalized genus of botanic gardens alien collections. Genus of the naturalized aliens are marked with stars. Most of the random sampled non-naturalized genus were having close phylogenetic relation with the naturalized genus (except for Piper, 1 species: *Piper aduncum*). Phylogenetic tree constructed in R (R Core Team 2013) using “brranching” package (Chamberlain 2020) and utilized phylogeny data from phylodiversity.net as phylogenetic tree reference.

**File S8.** R code used to calculate predicted density from naturalized aliens observations.

predsigma <- NULL

preddens23 <- NULL

w <- -0.005 #sqrt leaf area coefficient from detection model result (Junaedi et al. 2018)

y <- 0.044 #leaf area coefficient from detection model result (Junaedi et al. 2018)

z <- 0.112 #height coefficient from detection model result (Junaedi et al. 2018)

t <- -0.003 #shape coefficient from detection model result (Junaedi et al. 2018)

u <- -0.0001 #shape2 coefficient from detection model result (Junaedi et al. 2018) NDetected <- ab23$Ndetected[1:23]

Sh <- ab23$spSH[1:23]

spH <- ab23$spH[1:23]

spLA <- ab23$spLA[1:23]

spL <- ab23$spL[1:23]

Ls <- ab23$Ls[1:23]

MSA <- 8.86

MSh <- 6.39

pi <- 3.1416

for (i in 1:23) # for each of the 23 species

{

#apply hierarchical detectability model (Junaedi et al. 2018) to calculate predicted density

predsigma[i] <- exp(w*(sqrt((spLA[i]-MSA)*(spLA[i]-MSA)))

+ y*(spLA[i]-MSA)*(spLA[i]-MSA)

+ z*(spH[i]-2)

+ t*(sqrt((Sh[i]-MSh)*(Sh[i]-MSh)))

+ u*((Sh[i] - MSh)*(Sh[i] - MSh)))

#calculate predicted density

hapreddens24[i] <- NDetected[i] / (2 * Ls[i] * predsigma[i] * sqrt(pi/2))

}


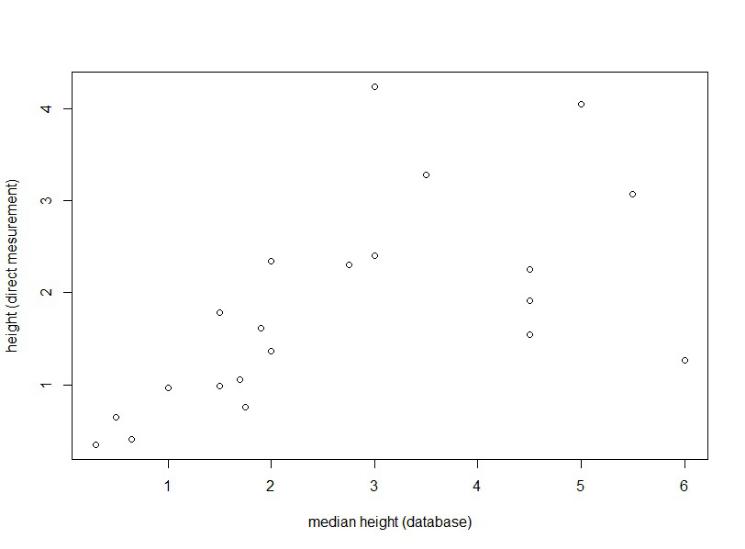


**Figure S9.** Scatter plot between height value from direct measurement versus height value (median) from available databases. If the minimum height value not stated in the database, we used the median value between zero and the maximum height that stated in the database.

**Table S10.** Comparison of r^2^ values of possible regression types from data plotted in Figure S7.

| **Fitted model** | **Equation** | **r^2^** |
| --- | --- | --- |
| Power | y = 0.9013x^0.6878^ | 0.6549 |
| Polynomial | y = -0.1901x^2^ + 1.552x - 0.4423 | 0.5509 |
| Logarithmic | y = 0.923ln(x) + 1.1508 | 0.4536 |
| Exponential | y = 0.7254e^0.269x^ | 0.4449 |
| Linear | y = 0.3873x + 0.7879 | 0.3548 |

**
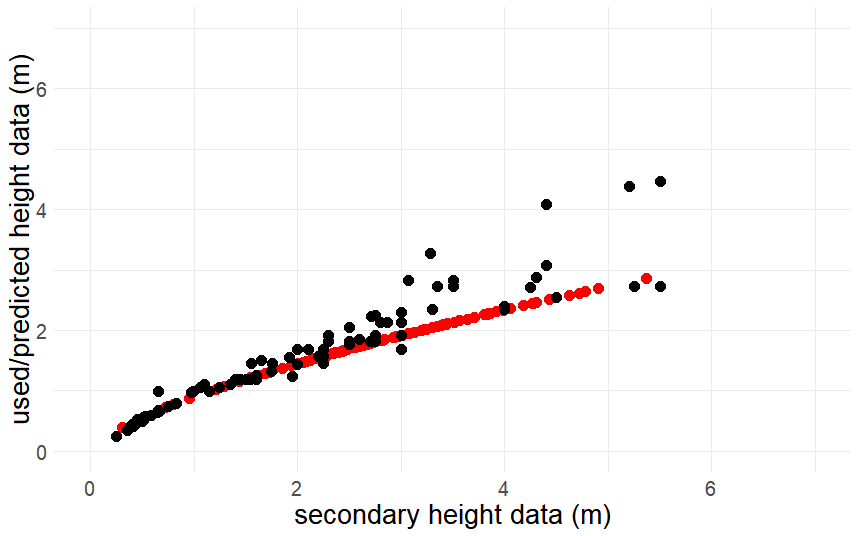
**

**FigureS11.** Scatter plot between height data from secondary sources (database) and predicted height data (black dots) and scatter plot between simulated height data and the model prediction (red dots)

**File S12.** R code used in Bayesian logistic regression analysis in this study (model 1)

blr78 <- read.csv("modelsla-22aug-2017ch5.csv")

blr78

summary (blr78)

blr78$time <- as.numeric(blr78$time)

#standardized SLA

sla1 <- blr78$sla

msla <- mean(sla1)

sdsla <- sd(sla1)

stdsla <- (sla1-msla)/(2*sdsla)

#standardized time

time1 <- blr78$time

mtime <- mean(time1)

sdtime <- sd(time1)

stdtime <- (time1-mtime)/(2*sdtime)

#standardized H

height1 <- blr78$height

mheight <- mean(height1)

sdheight <- sd(height1)

stdheight <- (height1-mheight)/(2*sdheight)

#standardized seedmass

sm1 <- blr78$sm

msm1 <- mean(sm1)

sdsm <- sd(sm1)

stdsm <- (sm1-msm1)/(2*sdsm)

y <- blr78$status

x <- stdsla

o <- stdtime

q <- stdheight

u <- stdsm

r2 <- blr78$dispersal2

sink(paste(getwd(),"/blognov17.txt",sep=""))

cat("

model {

for (i in 1:102)

{

logit(p[i]) <- a + sla*(x[i]) +

time*(o[i]) +

height*(q[i]) +

sm*(u[i]) +

andisp*r2[i]

y[i] ~ dbern(p[i])

}

a ~ dnorm(0, 1.0E-6)

sla ~ dnorm(0, 1.0E-6)

time ~ dnorm(0, 1.0E-6)

height ~ dnorm(0, 1.0E-6)

sm ~ dnorm(0, 1.0E-6)

andisp ~ dnorm(0, 1.0E-6)

sd ~ dnorm(0, 1.0E-6)

}

",fill=TRUE)

sink()

#JAGS statistical analysis

jags.data<-list(y=blr78$status,

x=stdsla,

o=stdtime,

q=stdheight,

u=stdsm,

r2=blr78$dispersal2)

inits<-function()(list(a=0, sla=0, time=0, height=0, sm=0, sd=0.1))

parameters<-c("a","sla","time","height","andisp","sm", "sd", "pSLA_animal","pSLA_notanimal","ptime_animal","ptime_notanimal","pheight_animal","pheight_notanimal")

ni<-50e3;nb<-ni/2;nt<-5;nc<-3

modelnov17<-jags(jags.data,inits,parameters,"blognov17.txt",n.chains=nc,n.thin=nt,n.iter=ni,n.burnin=nb)

save.image("blogrnov.RData")

modelnov17

**File S13.** R code for multiple regression analysis: predicted density as a function of traits (model 2)

ab24ins <- read.csv("abundancemodeldata-11september2017.csv")

ab24ins$timereal <- as.numeric(ab24ins$timereal)

#standardized SLA

sla2 <- ab24ins$sla

msla2 <- mean(sla2)

sdsla2 <- sd(sla2)

stdsla2<- (sla2-msla2)/(2*sdsla2)

#standardized H

height2 <- ab24ins$heightescapedata

mheight2 <- mean(height2)

sdheight2 <- sd(height2)

stdheight2 <- (height2-mheight2)/(2*sdheight2)

#standardized time

time2 <- ab24ins$timereal

mtime2 <- mean(time2)

sdtime2 <- sd(time2)

stdtime2 <- (time2-mtime2)/(2*sdtime2)

#standardized seedmass

sm2 <- ab24ins$sm

msm2 <- mean(sm2)

sdsm2 <- sd(sm2)

stdsm2 <- (sm2-msm2)/(2*sdsm2)

aa <- stdsla2

bb <- stdheight2

cc <- stdtime2

dd <- stdsm2

ee <- ab24ins$dispersal2

ff <- ab24ins$ldm

sink(paste(getwd(),"/bmlrnov17.txt",sep=""))

cat("

model

{

for (i in 1:23)

{

ff[i] ~ dnorm( mu[i], tau) #for density model (eq2)

mu[i] <- gg +

sla*(aa[i]) +

height*(bb[i]) +

time*(cc[i]) +

sm*(dd[i]) +

andis*(ee[i])

}

gg ~ dnorm(0, 0.001) #intercept

sla ~ dnorm(0, 0.001) #SLA

height ~ dnorm(0, 0.001) #height

time ~ dnorm(0, 0.001) #time

sm ~ dnorm(0, 0.001) #seedmass

andis ~ dnorm(0, 0.001) #animal dispersed

tau ~ dgamma(0.001, 0.001)

sigma2 <- 1/tau # regression standard deviation

}

",fill=TRUE)

sink()

#############################

### JAGS statistical analysis

#############################

jags.data24in3v<-list(ff=ab24ins$ldm,

aa=stdsla2,

cc = stdtime2,

bb=stdheight2,

dd=stdsm2,

ee=ab24ins$dispersal2)

inits<-function()(list(gg=0, sla=0, height=0, time=0, sm=0, andis=0, tau=0.1))

parameters<-c("gg","sla","height","time","sm", "andis")

ni<-50e3;nb<-ni/2;nt<-5;nc<-3

modelbmlr24nov17<-jags(jags.data24in3v,inits,parameters,"bmlrnov17.txt",n.chains=nc,n.thin=nt,n.iter=ni,n.burnin=nb)

save.image("bmlr24nov17.RData")

modelbmlr24nov17

**File S14.** R code used in multiple regression analysis of dispersal distance in this study (model 3)

ab24f <- read.csv2("spread_distance2020.csv")

ab24f <- read.csv("speciesdistancedata-aug2017.csv")

ab24f$lmediand <- log(ab24f$mediand)

dispersal2 <- ab24f$dispersal2

dlmed <- ab24f$lmediand

#standardized SLA

sla3 <- ab24f$sla

msla3 <- mean(sla3)

sdsla3 <- sd(sla3)

stdsla3<- (sla3-msla3)/(2*sdsla3)

#standardized time

time3 <- ab24f$timereal

mtime3 <- mean(time3)

sdtime3 <- sd(time3)

stdtime3 <- (time3-mtime3)/(2*sdtime3)

#standardized seedmass

sm3 <- ab24f$sm

msm3 <- mean(sm3)

sdsm3 <- sd(sm3)

stdsm3 <- (sm3-msm3)/(2*sdsm3)

#standardized height

height3 <- ab24f$height

mheight3 <- mean(height3)

sdheight3 <- sd(height3)

stdheight3 <- (height3-mheight3)/(2*sdheight3)

ll <- stdsla3

mm <- stdheight3

nn <- stdtime3

oo <- stdsm3

pp <- ab24f$dispersal2

qq <- dlmed

#model using log-transformed median species distance

sink(paste(getwd(),"/bmlr24fnov17.txt",sep=""))

cat("

model

{

for (i in 1:22)

{

qq[i] ~ dnorm( mu[i], tau ) #for distance to gardens model (eq3)

mu[i] <- hh +

sla*(ll[i]) +

height*(mm[i]) +

time*(nn[i]) +

sm*(oo[i]) +

andis*(pp[i])

}

hh ~ dnorm(0, 0.001) #intercept

sla ~ dnorm(0, 0.001) #sla

height ~ dnorm(0, 0.001) #height

time ~ dnorm(0, 0.001) #time

sm ~ dnorm(0, 0.001) #seedmass

andis ~ dnorm(0, 0.001) #animal dispersed

tau ~ dgamma(0.001, 0.001)

sigma2 <- 1/tau # regression standard deviation

}

",fill=TRUE)

sink()

##############################

### JAGS statistical analysis

##############################

jags.datanov17med<-list(qq=dlmed,ll=stdsla3,mm=stdheight3,nn=stdtime3,oo=stdsm3, pp=ab24f$dispersal2)

inits<-function()(list(hh=0, sla=0,height=0,time=0,sm=0,andis=0,tau=0.1))

parameters<-c("hh",”sla,”height”,”time”,”sm”, “andis”,”sigma”)

ni<-5e3;nb<-ni/2;nt<-5;nc<-3

bmlr24fnov17med<-jags(jags.datanov17med,inits,parameters,"bmlr24fnov17.txt",n.chains=nc,n.thin=nt,n.iter=ni,n.burnin=nb)

save.image("bmlr24fnov17med.RData")

bmlr24fnov17med

**
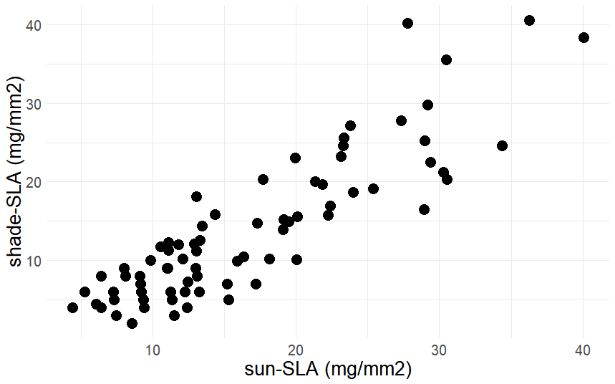
**

**Figure S15.** Scatter plot between measurement results of shade-SLA and sun-SLA of non-naturalized aliens

**Table S16.** Estimated regression coefficients of standardized predictors of naturalization probability (model 1) using two different SLA: sun-SLA (measured from sun exposed leaves) and shade-SLA (measured from shaded leaves). Analysis conducted through R using “lme4” package (Bates et al. 2015). Detected effects marked by asterisks.

| **Independent variable (standardized)** | **Estimated correlation coefficient** | **Standard error** | **Z value** | **Pr (>\|z\|)** | **AIC** |
| --- | --- | --- | --- | --- | --- |
| Sun-SLA | 6.27 | 1.50 | 4.17 | 0.000010 | 60.139 |
| Shade-SLA | 6.04 | 1.38 | 4.37 | 0.000012 | 58.147 |

**Table S17.** Estimated regression coefficients of standardized predictors of naturalization probability using generalized linear model (GLM) (botanic gardens (BG) as independent variable) and generalized linear mixed model (GLMM) (botanic gardens (BG) as random factor / site effects). Analysis conducted through R using “lme4” package (Bates et al. 2015). Detected effects marked by asterisks.

| **Model** | **Independent variable (standardized)** | **Estimated correlation coefficient** | **Standard error** | **Z value** | **Pr (>\|z\|)** | **AIC** |
| --- | --- | --- | --- | --- | --- | --- |
| **GLM** | **SLA*** | 6.24 | 1.46 | 4.26 | 0.00005 | 63.53 |
|  | H | -0.96 | 1.12 | -0.86 | 0.39 |  |
|  | **DM*** | -2.05 | 1.02 | -2.01 | 0.04 |  |
|  | SM | -0.26 | 1.71 | -0.15 | 0.88 |  |
|  | MRT | 1.26 | 1.46 | 0.863 | 0.39 |  |
|  | BG-Baturraden | -0.29 | 1.44 | -0.21 | 0.84 |  |
|  | BG-Cibodas | -0.05 | 1.41 | -0.04 | 0.97 |  |
|  | BG-Kuningan | -17.33 | 2190.28 | -0.01 | 0.99 |  |
| **GLMM** | **SLA*** | 6.04 | 1.38 | 4.37 | 0.00001 | 60.1 |
|  | H | -1.07 | 1.10 | -0.97 | 0.33 |  |
|  | **DM*** | -1.99 | 1.02 | -1.97 | 0.05 |  |
|  | SM | -1.29 | 1.06 | -1.22 | 0.22 |  |
|  | MRT | 1.42 | 0.82 | 1.73 | 0.08 |  |

**Table S18.** Estimated regression coefficients of standardized predictors for the statistical models of probability of naturalization (model 1), density of aliens (model 2), and spread distance from gardens (model 3) of standardized variable values. Values provided are the mean and standard deviation of the posterior distributions, and 95% credible intervals. Detected effects marked with asterisks (*).

| **Model** | **Variables** | **Standardized model**  **(variables with standardized value)** | | |
| --- | --- | --- | --- | --- |
|  |  | **mean** | **sd** | **95% CI** |
| **Probability of** | ***SLA** | **7.03** | **1.58** | **[4.30, 10.51]** |
| **naturalization** | H | -1.45 | 1.17 | [-3.97, 0.59] |
|  | ***DM** | **-2.32** | **1.11** | **[-4.65, -0.33]** |
|  | ***SM** | **-2.32** | **1.65** | **[-6.29,-0.01]** |
|  | ***MRT** | **1.73** | **0.92** | **[0.01, 3.62]** |
| **Log_10_ (density)** | SLA | 0.47 | 0.45 | [-0.43,1.36] |
|  | H | -0.47 | 0.43 | [-1.31,0.38] |
|  | DM | -0.45 | 0.49 | [-1.41,0.53] |
|  | SM | 0.30 | 0.47 | [-0.61,1.25] |
|  | MRT | 0.59 | 0.46 | [-0.32,1.50] |
| **Log_10_** | SLA | 1.06 | 0.59 | [-0.09,2.19] |
| **(spread distance)** | H | 0.27 | 0.59 | [-0.93,1.41] |
|  | ***DM** | **-1.21** | **0.63** | **[-2.46,-0.03]** |
|  | SM | 0.42 | 0.62 | [-0.84,1.71] |
|  | MRT | 0.59 | 0.60 | [-0.59,1.69] |


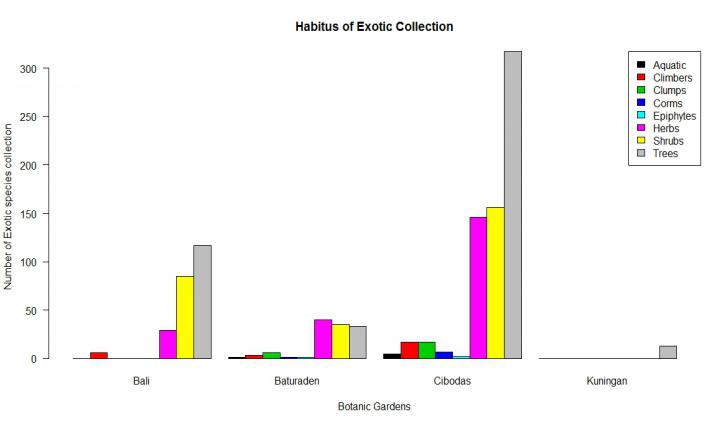
**Figure S19.** Total number of alien plant species collection from Bali Botanic Gardens, Baturraden Botanic Gardens, Cibodas Botanic Gardens and Kuningan Botanic Gardens based on their growth form (Collins Dictionary 2020).


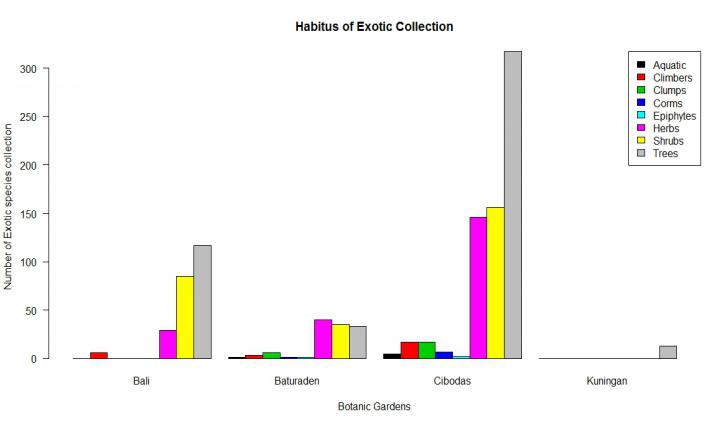

Supplement: Supplementary file 1 [file ECE3-11-3808-s004.docx]
